# Genome Sequencing Unveils the Role of Copy Number Variants in Hearing Loss and Identifies Novel Deletions With Founder Effect in the DFNB1 Locus

**DOI:** 10.1155/2024/9517114

**Published:** 2024-08-06

**Authors:** Zibin Lin, Jiale Xiang, Xiangzhong Sun, Nana Song, Xiaozhou Liu, Qinming Cai, Jing Yang, Haodong Ye, Jiangfan Xu, Hongfu Zhang, Jiguang Peng, Yu Sun, Zhiyu Peng

**Affiliations:** ^1^ College of Life Sciences University of Chinese Academy of Sciences, Beijing 100049, China; ^2^ BGI Genomics, Shenzhen 518083, China; ^3^ Hunan Provincial Key Laboratory of Regional Hereditary Birth Defects Prevention and Control Changsha Hospital for Maternal & Child Health Care Affiliated to Hunan Normal University, Changsha, China; ^4^ Department of Otorhinolaryngology Union Hospital of Tongji Medical College Huazhong University of Science and Technology, Wuhan 430022, China

## Abstract

Sensorineural hearing loss is a prevalent disorder with significant genetic involvement, which is often challenging to diagnose due to genetic heterogeneity. Exome sequencing (ES) has been a standard diagnostic tool for sensorineural hearing loss, but its limitations in detecting copy number variants (CNVs) and intronic variants have prompted the exploration of genome sequencing (GS) for improved diagnostic yield. We conducted GS on 46 hearing loss families with previously negative ES results and an additional cohort of 36 patients with a monoallelic pathogenic variant in *GJB2* (the most common deafness gene). Additionally, the impact of a previously unrecognized novel 125-kb deletion in the DFNB1 locus on *GJB2* expression was assessed using quantitative polymerase chain reaction (qPCR), and haplotype analysis was performed to characterize the deletion. GS diagnosed eight cases (17%, 8/46) in the ES-negative cohort, primarily attributed to CNVs (6/8). Notably, a previously unrecognized 125 kb deletion in the DFNB1 region was identified, affecting *GJB2* expression and characterizing it as a founder effect in East Asian. In 47 patients with a monoallelic *GJB2* variant, 15% (95% CI, 7.4%–28%) were diagnosed with DFNB1 deletions. Analysis of the gnomAD database revealed the prevalence and ethnic diversity of DFNB1 deletions, with the novel 125 kb deletion emerging as a prominent pathogenic variant in East Asian, non-Finnish European, and admixed American populations. Our study highlights the utility of GS in diagnosing sensorineural hearing loss. The identification of DFNB1 deletions underscores their significant contribution to hearing loss etiology, advocating for their inclusion in routine diagnostic testing. We propose GS as a primary genetic testing approach for patients with hearing loss, offering comprehensive genomic analysis and the potential for improved diagnostic accuracy.

## 1. Introduction

Hearing loss, a prevalent sensorineural disorder, has an incidence of approximately 0.1%–0.3% among newborns [1, 2]. It was estimated that genetic causes account for up to 60% of patients with congenital bilateral hearing loss [3]. To date, over 100 genes have been associated with nonsyndromic hearing loss, while more than 400 genes are reported to be linked with syndromic hearing loss [4]. Both single nucleotide variants (SNVs) and copy number variants (CNVs) are common contributors to hearing loss [5]. Statistically, around 20% of hearing loss cases might be attributed to CNVs [6]. Genetic heterogeneity poses challenges in the molecular diagnosis of hearing loss.

Exome sequencing (ES) has been widely used to identify the genetic cause of hearing loss due to its capability of identifying SNVs spanning human exomes [7]. In recent years, the advance of analytic pipelines enables CNV calling from ES data. However, ES has inherent limitations in the detection of CNVs. For example, the *STRC* gene is one of the most common hearing loss genes having a high incidence of CNVs [6]. Due to the existence of a highly homologous pseudogene (*pSTRC1*), ES has the disadvantage of error-prone capture biases for *STRC* and does not reliably detect *STRC* variants [8, 9]. Genome sequencing (GS) provides more even and unbiased coverage than ES [10]. Recently, our group reported high sensitivity (100%) and specificity (98.8%) in detecting *STRC* variants from GS data [11].

In addition, ES generally failed to detect variants involving the intronic and intergenic regions [12]. For instance, *GJB2* is the most common deafness gene causing autosomal recessive nonsyndromic hearing loss (DFNB1A, OMIM #220290). The DFNB1 deletion, located at the upstream of *GJB2*, was reported to regulate the expression of *GJB2* [13]. Previous studies identified six types of DFNB1 deletions in the European population: del(*GJB6*-D13S1830), del(*GJB6*-D13S1854), del(179 kb), del(131 kb), del(>920 kb), and del(101 kb) [13, 14]. Interestingly, the DFNB1 deletion has not yet been reported in the East Asian population.

GS is considered a preferred approach for genetic diagnosis. Studies have evaluated the diagnostic performance between GS and ES across various genetic diseases, demonstrating its advantage in detecting intronic variants, noncoding regulating regions, and CNVs [12]. Currently, there has been no study systematically examining the utility of GS in the diagnosis of hearing loss.

In this study, we conducted GS on 46 families experiencing hearing loss, despite initially obtaining negative results with ES. The identification of new diagnostic cases was primarily attributed to CNVs. Notably, we discovered a previously unrecognized 125 kb deletion in the DFNB1 region, emphasizing its significant role in the genetic etiology of individuals with hearing loss in the Chinese population. Lastly, we highlighted the importance of the DFNB1 deletions across ethnically diverse populations by analyzing the gnomAD v4.1.0 database with the availability of the structural variant dataset.

## 2. Materials and Methods

### 2.1. Recruitment

This study involved two patient cohorts. Cohort 1 comprised 46 families with hearing loss who had previously tested negative through ES. Probands with bilateral nonsyndromic sensorineural hearing loss were recruited, whereas patients with unilateral, conductive, or mixed hearing loss were excluded. Cohort 2 consisted of 36 patients with ES-negative results but carrying a monoallelic pathogenic *GJB2* variant. This cohort was recruited to investigate the impact of DFNB1 deletions in individuals with a monoallelic pathogenic *GJB2* variant. Both Cohort 1 and Cohort 2 were recruited from Union Hospital of Tongji Medical College of Huazhong University of Science and Technology.

### 2.2. Ethics Approval and Patient Consent

The study was approved by the institutional review boards of Tongji Medical College of Huazhong University of Science and Technology (2022-S041) and BGI (22089). Written informed consent was obtained from all the individuals participating in this study or their legal guardians, ensuring compliance with ethical standards.

### 2.3. Sequencing and Bioinformatic Analysis

Genomic DNA extraction was performed from peripheral blood or saliva samples using a universal DNA extraction kit (Magen, Guangzhou, China). GS was conducted on the DNBSEQ-T7 platform (BGI-Wuhan, Wuhan, China) with 100 bp paired-end reads, following established protocols [15]. The raw sequencing data underwent processing, filtering, and quality control based on the following criteria: (1) the average depth after duplication removal should be equal to or greater than 40×; (2) at least 95% of the genome sequence should be covered at a depth of 20× or more. Subsequently, the raw sequence data were aligned to the human genome reference sequence (GRCh37/hg19) using the Burrows–Wheeler Aligner best practices pipeline [16]. SNVs and insertion/deletion (Indel) variants were identified utilizing the Genome Analysis Tool Kit [17]. CNVs were detected using ExomeDepth, CNVnator, and cn.mops [18–20].

### 2.4. Variant Interpretation and Confirmation

The pathogenicity assessment of SNVs, Indels, and CNVs adhered to the guidelines for the interpretation of sequence variants for genetic hearing loss [21] and the ACMG-AMP CNV interpretation guideline [22], respectively. Pathogenic or likely pathogenic SNVs/Indels were validated through Sanger sequencing. CNVs were confirmed using quantitative polymerase chain reaction (qPCR) or multiplex ligation–dependent probe amplification. The breakpoints of CNVs were further validated using Sanger sequencing.

### 2.5. The Impact of the Novel del(125 kb) on the *GJB2* Expression

Buccal epithelium samples were collected using Cytosoft brushes. RNA extraction was carried out using a high-purity total RNA extraction kit (Proteinssci, Shanghai, China). Subsequently, the purified total RNA underwent reverse transcription into cDNA using the PrimeScript™ FAST RT reagent Kit with gDNA Eraser (Takara, Shiga, Japan). qPCR was performed to assess the expression of *GJB2*. TB Green Premix Ex Taq™ II (Takara, Shiga, Japan) was employed for qPCR, and qPCR analysis was conducted on the CFX Connect Real-Time PCR Detection System (Bio-Rad, United States).

### 2.6. Haplotype Analysis for del(125 kb)

Haplotypes within a 1 Mb region surrounding the del(125 kb) were characterized utilizing a set of 21 single nucleotide polymorphisms (SNPs). SNP selection was based on allelic frequency data obtained from the ChinaMap [23]. The haplotype analysis was conducted on four patients with the del(125 kb) variant and their respective parents. Haplotypes were established through read-based SNP phasing utilizing GS data. For four individuals whose parents' samples were not available, we employed long-read sequencing to establish the haplotype. The allele frequency of SNPs derived from ChinaMap and this study was analyzed with Fisher's exact test, and those with a significant difference were regarded as SNPs with linkage disequilibrium. In order to estimate the linkage disequilibrium region, we adopted a criteria whereby a region was not considered to be linkage disequilibrium if two consecutive SNPs showed *p* > 0.05 [24].

## 3. Results

### 3.1. Diagnostic Yield of GS in ES-Negative Families

**Figure 1:**
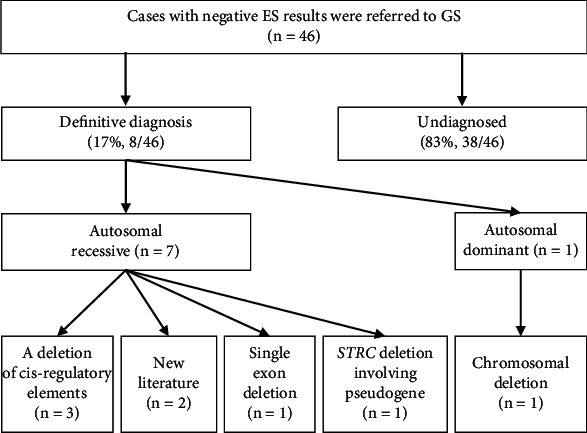
Diagnostic yield of GS in ES-negative families. GS enhanced the overall diagnostic yield by 17% (8/46), attributing to diagnoses originating from the deletion of *cis*-regulatory regions of a functional gene (*n* = 3), new literature (*n* = 2), single exon deletion (*n* = 1), *STRC* deletion involving pseudogene (*n* = 1), and chromosomal deletion (*n* = 1), with 75% (6/8) of them being copy number variants.

Of 46 families with ES-negative results (the demographic characteristics of 46 probands are shown in Table S1), GS diagnosed eight cases (17%, 8/46) (Figure 1). Specifically, six cases were contributed to CNVs, whereas the remaining two cases (Cases #26 and #29) were contributed to supportive evidence from newly published literature (Table 1 and Table S2).

**Figure 2:**
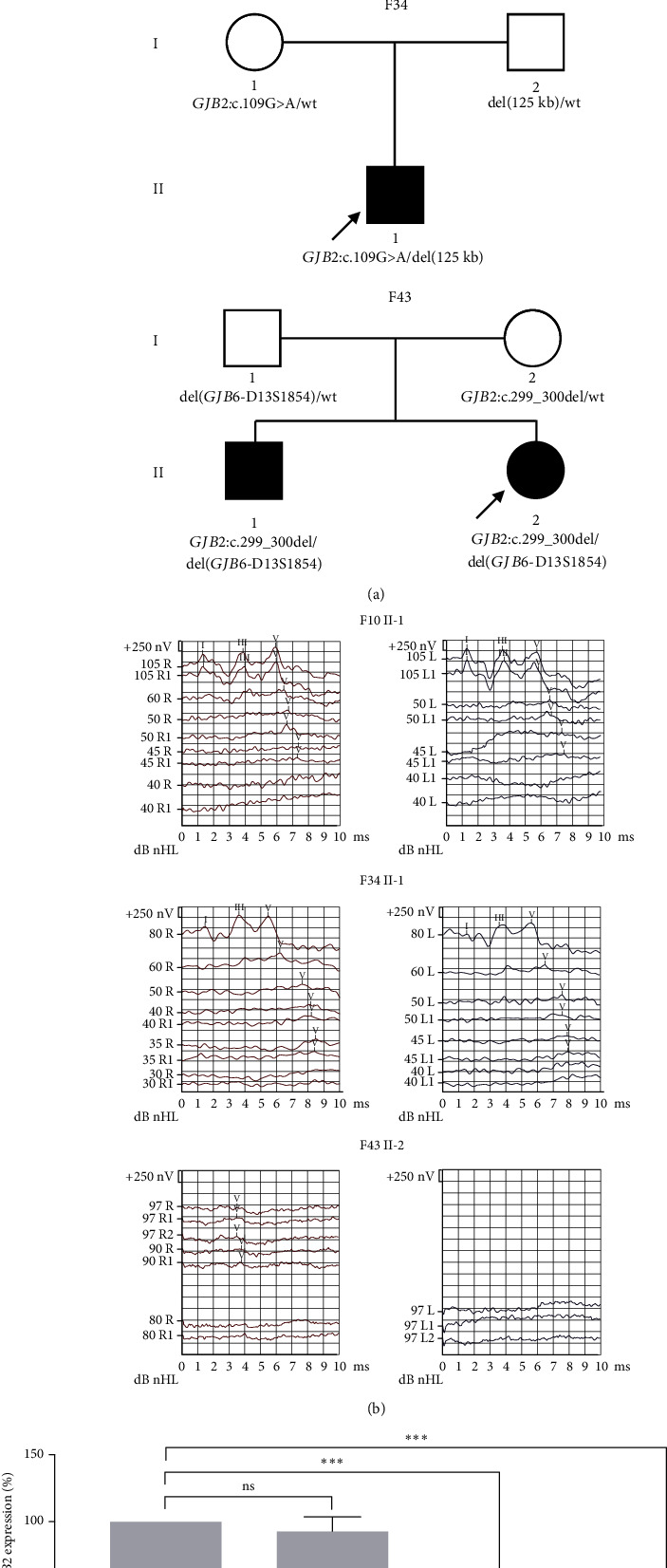
Identification of DFNB1 deletions in probands with a monoallelic *GJB2* variant. (a) Pedigree diagrams depicting families with negative results from ES. Probands from Family 10 (F10) and 34 (F34) carry a novel del(125 kb) *in trans* with *GJB2* c.109G>A, while the proband from Family 43 (F43) harbors del(*GJB6*-D13S1854) *in trans* with *GJB2* c.299_300del. (b) Audiograms of the three probands. Probands from F10 and F34 exhibit mild-to-moderate hearing loss, while the proband from F43 presents with profound hearing loss. Waves I, III, and V are clinically important waveforms of auditory brain response. The hearing threshold is determined by Wave V. (c) Relative expression levels of *GJB2* in buccal epithelium assessed by RT-qPCR. *GJB2* expression, normalized to *β*-actin, is shown for different samples. RT-qPCR reactions were performed in triplicate for each sample, and the data are expressed as mean ± SD. Significance is denoted by ^∗∗∗^*p* < 0.001. Abbreviations: ns, not significant; wt, wild type. The *GJB2* transcript is denoted as NM_004004.6.

Of the six cases associated with CNVs, three cases (#10, #34, and #43) harbored a deletion involving the upstream region of the *GJB2* gene (DFNB1 deletions); two of them (#10 and #34) shared an identical novel 125 kb deletion, and the other case (#43) had a reported deletion del(*GJB6*-D13S1854) (Table 1 and Figure 1). The three cases were accompanied by a monoallelic pathogenic *GJB2* variant *in trans* configuration (Figure 2(a)). Phenotypically, two probands carrying del(125 kb) and *GJB2* c.109G>A (p.Val37Ile) exhibited mild-to-moderate hearing loss (F10 II-1 and F34 II-1), while the other proband (F43 II-1) carrying del(*GJB6*-D13S1854) and *GJB2* c.299_300del (p.His100ArgfsTer14) presented with profound hearing loss (Figure 2(b)). In addition, qPCR was conducted to assess the effect of the novel del(125 kb) on the expression of *GJB2* in Family 34. The proband and his father, who harbored del(125 kb), exhibited a significantly decreased *GJB2* expression compared with his mother and the control sample (Figure 2(c)).

In family 28, the proband was identified with a 6.8 kb homozygous deletion spanning Exon 87 in the *ADGRV1* gene, while the parents exhibited a heterozygous deletion, as revealed by GS. Interestingly, upon reanalysis of the ES data, the homozygous deletion in the proband was successfully identified, whereas the heterozygous deletion in the parents was not detected. Given the limitations of detecting single exon CNVs using ES data and the extremely low likelihood of de novo homozygous CNVs, the homozygous deletion was prioritized as a false positive result through pedigree analysis in the clinical ES test and was not reported (Table 1 and Figure S1).

In Family 42, the diagnosis of Smith–Magenis syndrome was confirmed through the identification of a 3.7 Mb deletion in chromosome 17p11.2 (Table 1 and Figure S2). The breakpoints of this deletion were unnoticed as the probes used in ES did not cover this specific region. In Family 44, a homozygous deletion involving *STRC* and *CATSPER2*, leading to deafness–infertility syndrome (OMIM #611102), was identified through GS utilizing our newly developed analytic pipeline [11]. ES has the disadvantage of error-prone capture biases and false mapping of highly homologous pseudogenes, making it challenging to analyze these regions accurately.

Overall, even though eight probands initially exhibited nonsyndromic hearing loss, GS revealed that five of them (5/8) actually had syndromic hearing loss, which was recognized as nonsyndromic hearing loss mimics. Given that certain phenotypes may manifest later or be specific to a particular gender, these probands were subsequently referred to specialists for precise medical management.

### 3.2. The Contribution of DFNB1 Deletions for Patients Harboring a Monoallelic *GJB2* Variant

Among the 46 probands with negative ES results in Cohort 1, 11 were found to carry a monoallelic *GJB2* variant. Interestingly, three of these patients (27%) were identified to have DFNB1 deletions through GS.

To further investigate the involvement of DFNB1 deletions in patients with a monoallelic *GJB2* variant, an additional 36 patients were recruited as Cohort 2. As a result, 11% (4/36) of cases exhibited an identical heterozygous del(125 kb) (Cases P66, P71, P76, and P82 in Table S3), located in the upstream of *GJB2*. Among them, three cases carrying the *GJB2* c.109G>A variant presented with mild-to-moderate hearing loss, while one case carrying the *GJB2* c.235del (p.Leu79CysfsTer3) variant had profound hearing loss.

**Figure 3:**
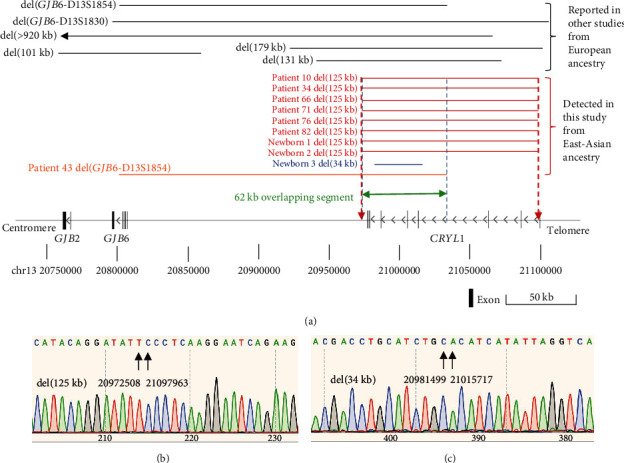
Overview of DFNB1 deletions on chromosome 13q12. (a) Schematic representation depicting the positions of DFNB1 deletions. Black lines denote types of DFNB1 deletions previously reported in studies involving individuals of European ancestry. Red, blue, and orange lines represent three distinct DFNB1 deletions identified in the East Asian population in this study. Specifically, a novel del(125 kb) was detected in six patients and two newborns, del(*GJB6*-D13S1854) was identified in one patient, and del(34 kb) was found in one newborn. The shared segment among all pathogenic DFNB1 deletions is approximately 62 kb, situated at chr13:g.20972509_21034768 (GRCh37). (b) Breakpoints of del(125 kb) are consistent across all eight cases, as confirmed by Sanger sequencing. The 125-kb deletion is positioned at chr13:g.20972509_21097962 (GRCh37). (c) The 34 kb deletion is located at chr13:g.20981500_21015716 (GRCh37).

Combining patients with a monoallelic *GJB2* variant from Cohort 1 (*n* = 11) and Cohort 2 (*n* = 36), the total number reached 47. Among them, 15% (7/47, 95% CI, 7.4%–28%) could be diagnosed due to DFNB1 deletions. Of the seven patients, six carried del(125 kb), and one carried del(*GJB6*-D13S1854) (Figure 3). All seven patients exhibited prelingual nonsyndromic hearing loss and did not pass the newborn hearing screening. Patients carrying the *GJB2* nontruncating variant (*GJB2* c.109G>A) showed mild-to-moderate hearing loss, whereas those with the *GJB2* truncating variants (*GJB2* c.235del or *GJB2* c.299_300del) presented with profound hearing loss (Table S3).

### 3.3. The Prevalence of DFNB1 Deletions in the General Populations

Considering the high frequency of DFNB1 deletions in hearing loss patients, we aimed to explore their prevalence in the general population. Specifically, we investigated the carrier frequency of DFNB1 deletions in 496 healthy newborns from our in-house newborn genome screening project database. As a result, two newborns carried the heterozygous del(125 kb), and another newborn carried a novel heterozygous del(34 kb). Overall, the carrier frequency of DFNB1 deletions was estimated to be 0.6% (3/496, 95% CI, 0.21%–1.8%) within the Chinese population.

**Figure 4:**
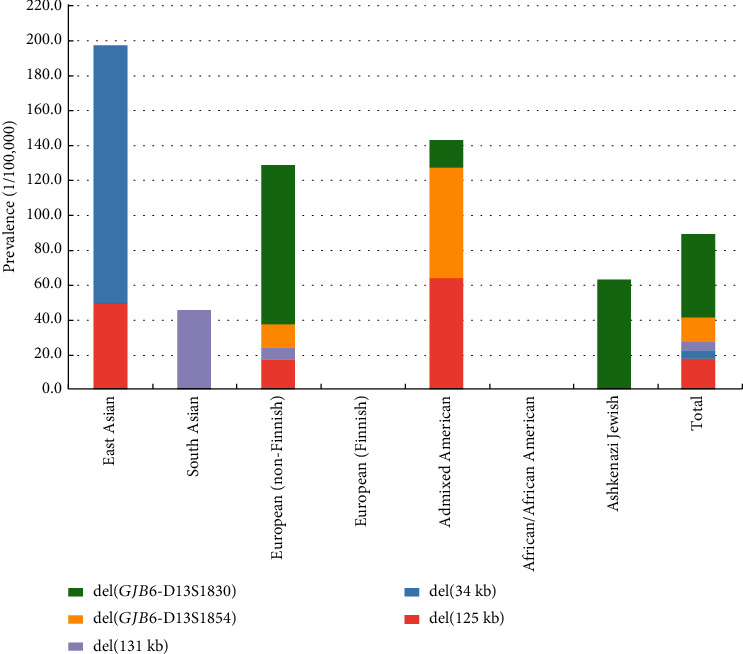
Frequency of DFNB1 deletions at the upstream of *GJB2* in gnomAD database across various ethnic populations. del(101 kb) and del(179 kb) were excluded from the illustration as they were not identified in the gnomAD database.

Then, we analyzed 63,046 genomes in gnomAD (v4.1.0 with the availability of a structural variant dataset) for DFNB1 deletions (Figure 4). Of note, DFNB1 deletions were uncovered in the African/African American and Finnish populations but prevalent among all other ethnic populations, ranging from 45.4/100,000 in South Asian to 197.3/100,000 in East Asian. Interestingly, the types of DFNB1 deletions varied across different ethnic populations. del(*GJB6*-D13S1830) was prevalent in the non-Finnish European (91.4/100,000) and Ashkenazi Jewish (62.9/100,000) and absent in South Asian and East Asian. del(131 kb) was enriched in South Asian, and del(34 kb) was exclusively detected in East Asian. The del(125 kb), the novel DFNB1 deletion detected in this study, had a prevalence of 16.9/100,000, 49.3/100,000, and 63.5/100,000 in the non-Finnish European, East Asian, and admixed American, respectively. del(101 kb) and del(179 kb) were not illustrated as they were not found in the gnomAD database.

### 3.4. The Breakpoints and Evolutionary Origin of the Novel del(125 kb)

Through Sanger sequencing, the location of del(125 kb) was chr13:g.20972509_21097962 (GRCh37) (Figure 3(b)). It was identical to the 125-kb deletion recorded in the gnomAD database. The proximal breakpoint of del(125 kb) was located downstream of *CRYL1*, while the distal breakpoint was within Intron 1 of *CRYL1* (Figure 3(a)). Around 120 kb of the 125 kb sequence (96%) was overlapped with the *CRYL1* gene. Sanger sequencing further confirmed that the breakpoint junctions were identical across all individuals with del(125 kb). Subsequently, the evolutionary origins of del(125 kb) were analyzed through haplotype analysis. Analyzing 21 SNPs linked to del(125 kb), it was observed that all chromosomes carrying del(125 kb) shared a core haplotype ranging from rs4238153 to rs7995798. The linkage disequilibrium range was approximately 240 kb for del(125 kb) (Table S4).

## 4. Discussion

GS has been proposed as an effective diagnostic test for identifying genetic abnormalities in monogenic patients. However, studies have not evaluated its additional yield in individuals with hearing loss. In this study, we examined 46 patients with nonsyndromic sensorineural hearing loss who had previously tested negative for ES. The overall diagnostic yield of GS in this cohort was 17% (8/46). Notably, 75% of the new diagnoses were associated with CNVs. Our study also revealed a previously unrecognized 125-kb deletion at the upstream of *GJB2*. This deletion accounts for 15% of the genetic etiology in patients carrying a monoallelic pathogenic *GJB2* variant. Finally, DFNB1 deletions exhibited a noticeable prevalence but varied types across different ethnic populations, emphasizing the importance of this region in hearing loss globally.

In the context of hearing loss patients, the hierarchical genetic testing strategy is generally recommended owing to the genetic heterogeneity [25, 26]. The first tier of this strategy involves targeted assays concentrating on high-yield genes, such as *GJB2* analysis, DFNB1, and *STRC* deletion analysis. Subsequently, samples that yield nondiagnostic results in the first tier proceed to the second tier, where the remaining deafness genes are assessed through ES. It is important to note that this sequential approach may lead to a diagnostic odyssey for some patients. In light of the demonstrated efficacy of GS in this study, there is potential for streamlining and simplifying the current diagnostic workflow through the early adoption of GS. This strategic integration could result in a more efficient and comprehensive approach to hearing loss, potentially expediting the diagnostic process.

GS offers distinct advantages in the diagnosis of monogenic disorders characterized by allelic heterogeneity, particularly those involving CNVs, such as hearing loss. In our study, CNVs played a predominant role, constituting 75% of newly identified diagnoses. These CNVs were classified into four groups: single exon deletion (Case #28 with Exon 87 deletion in *ADGRV1*), gene deletion involving a pseudogene (Case #44 with a homozygous deletion involving *STRC* and *CATSPER2*), chromosomal deletion spanning multiple functional genes (Case #42 with a 3.7 Mb chromosomal deletion), and the deletion of *cis*-regulatory regions of a functional gene (Cases #10, #34, and #43 with a 125 kb deletion downregulating the expression of *GJB2*). The unbiased coverage across the genome empowers GS to detect these CNVs [10]. Furthermore, GS provides precise breakpoints for CNVs, offering valuable insights into the underlying etiology and facilitating the assessment of CNV pathogenicity [27–29].

It is worth noting that DFNB1 deletions, including the unrecognized novel del(125 kb), accounted for 38% (3/8) of new diagnoses among patients with negative ES results. Individuals experiencing hearing loss carried a heterozygous del(125 kb) in combination with a monoallelic pathogenic *GJB2* variant *in trans* configuration, whereas their parents who carried either a heterozygous del(125 kb) or a pathogenic *GJB2* variant exhibited no hearing impairment. Furthermore, the del(125 kb) was shown to be associated with a significant reduction in *GJB2* expression, as confirmed by qPCR. This finding aligns with previously reported DFNB1 deletions, where the elimination of *GJB2* expression occurred through *cis*-regulatory elements [30, 31]. The segregation and expression data strongly support the role of the 125 kb deletion as a pathogenic factor in *GJB2*-related hearing loss.

To date, several types of DFNB1 deletions have been documented (Table S5). These include del(*GJB6*-D13S1830) (309 kb deletion involving the *GJB6* gene), del(*GJB6*-D13S1854) (232 kb deletion involving the *GJB6* gene), a 131 kb deletion telomeric to *GJB2* and *GJB6*, a 101 kb deletion involving *GJB2*, a 179 kb deletion with undefined breakpoints detected by ddPCR, and del(>920 kb) [6]. Utilizing structural variant data from the gnomAD database [32], these deletions exhibit marked variability in frequency and ethnic distribution. Notably, the newly identified pathogenic 125 kb deletion is not only prevalent in East Asian populations but also observed in non-Finnish European and admixed American populations. These findings underscore the significance of incorporating this regulatory region into routine clinical tests for the molecular diagnosis of hearing loss across diverse ethnic groups.

Currently, the detection of DFNB1 deletions has been lacking in East Asian individuals with a monoallelic pathogenic *GJB2* variant, primarily due to the prevalent use of targeted sequencing tests that inadequately cover the upstream region of *GJB2* [33, 34]. In this study, we elucidate that 15% of individuals exhibiting negative ES results harboring a monoallelic pathogenic *GJB2* variant can be diagnosed through deletions situated upstream of *GJB2*. Notably, all individuals with del(125 kb) shared a core haplotype, leading us to consider del(125 kb) as a founder effect variant in the East Asian population, akin to the role of del(*GJB6*-D13S1830) in the European population. Approximately 20% of individuals with a monoallelic pathogenic *GJB2* variant could be diagnosed owing to the presence of del(*GJB6*-D13S1830), with the diagnostic yield exhibiting variability across different countries [14]. Furthermore, our study discloses that 0.4% (2/496) of unaffected newborns in the Chinese population carry a heterozygous del(125 kb), which was comparable to the carrier frequency of del(101 kb) among individuals with normal hearing in Russia (0.5%) [35]. The prevalence of del(125 kb) among unaffected individuals supported it as the fifth most common pathogenic variant associated with hearing loss in China, following c.109G>A, c.235del of *GJB2*, c.919-2A>G of *SLC26A4*, and c.299_300del (p.His100ArgfsTer14) of *GJB2*, according to previously reported studies [36, 37].

The refinement of the *cis*-regulatory element of *GJB2* is of importance in elucidating the mechanisms of *GJB2*-related nonsyndromic hearing loss, which represents the most prevalent form of hearing loss. Based on all reported upstream pathogenic DFNB1 deletions, the overlapping segment spans 95 kb [13]. Incorporating the novel 125 kb deletion explored in this study, the refined overlapping segment is reduced to 62 kb, representing a notable decrease of 35%. Remarkably, 57 out of this 62 kb segment (91%) is situated within the *CRYL1* gene. Recent investigations have demonstrated that DNA elements interacting with *GJB2* exhibit *cis*-regulatory activities, localized within *CRYL1* [38]. Notably, the impactful elements influencing the promoter activity of *GJB2* span 1043 bp (chr13:20,993,543-20,994,585) [39], fully encompassed by the novel 125 kb deletion (chr13:20,972,509-21,097,962) identified in this study. Additionally, it is noteworthy that the 62 kb segment does not overlap with the *GJB6* gene, contrary to previous considerations implicating *GJB6* in *GJB2* expression due to del(*GJB6*-D13S1830) and del(*GJB6*-D13S1854). Subsequent functional studies of *GJB6* [40], coupled with the identification of DFNB1 deletions (131 kb, 125 kb, and 179 kb) that preserve the integrity of *GJB6*, have led to the reevaluation of *GJB6* as a hearing loss gene. Recent decisions by the ClinGen Hearing Loss Expert Panel further refute any association of *GJB6* with nonsyndromic hearing loss.

One limitation of this study was that the pathogenicity of the 34 kb deletion (chr13:20,981,500-21,015,716) remains elusive. This unreported deletion was identified in a single individual from our in-house unaffected newborn genome screening cohort and exhibited a prevalence of 148/100,000 in East Asians according to the gnomAD database, higher than any other documented DFNB1 deletions (Figure 4). While the lack of segregation or functional data currently restricts our understanding, it is noteworthy that the 34 kb region fully encompasses the previously mentioned 1043 bp *cis*-regulatory element [39]. This observation suggests a potential role for the 34-kb deletion in hearing loss. Nonetheless, additional investigations are warranted to elucidate its pathogenicity. In addition, a growing number of complex structural variants associated with hearing loss have been reported [41]. Due to short read lengths, short-read GS has limitations in identifying cryptic structural variants, sequencing repetitive regions, and distinguishing highly homologous genomic regions, which results in a risk of missed diagnoses. However, long-read GS generates ultralong sequencing reads (>10 kb on average) and avoids PCR-related biases, demonstrating better capability in characterizing the aforementioned complex structural variants [42]. Further research on the clinical value of long-read GS for the molecular diagnosis of hearing loss is highly necessary.

## 5. Conclusions

In summary, this study underscores the effectiveness of GS in diagnosing nonsyndromic sensorineural hearing loss, with a notable contribution from CNVs. Considering the substantial impact of DFNB1 deletions and their diverse distribution across ethnicities, we advocate for the inclusion of DFNB1 deletions in routine diagnostic testing. Moreover, we propose GS as a primary genetic testing approach for diagnosing patients with hearing loss.

## Figures and Tables

**Table 1:** New diagnostic cases achieved by genome sequencing.

**Family ID**	**Sex**	**Disorder**	**Gene**	**Zygosity**	**HGVS variant**	**Variant type**	**Reference (PMID)**	**ACMG/AMP criteria**	**Classification**	**Reasons for undiagnosed by clinical ES**
10, 34	Male	DFNB1A	*GJB2*	Het	NM_004004.6:c.109G>A	SNV	10982180	PS4, PM3, PP1_Strong	P	A deletion of the upstream regulatory elements of *GJB2* Uncovered by exome probes
			—	Het	NC_000013.10:g.20972509_21097962del	CNV	This study	NA	—	
43	Female	DFNB1A	*GJB2*	Het	NM_004004.6:c.299_300del	Indel	26043044	PVS1, PM2_Supporting, PM3_VeryStrong	P	
			—	Het	NC_000013.10:g.20802727_21034768del del(*GJB6*-D13S1854)	CNV	15994881	NA	—	
26, 29	Male	Perrault syndrome 4	*LARS2*	Comp het	NM_015340.4:c.1886C>T	SNV	23541342	PM2, PM3, PP1, PP4	LP	New literature
					NM_015340.4:c.1661T>C	SNV	This study	PM2, PM3_Strong	LP	
28	Male	Usher syndrome, Type IIC	*ADGRV1*	Hom	NC_000005.9:g.90397214_90404089del (Exon 87)	CNV	This study	PVS1, PM2, PM3, PP1	P	The heterozygous single exon deletion is undetectable by ES
42	Female	Smith–Magenis syndrome	*RAI1* etc.	Het	NC_000017.10:g.16748785_20420450del	CNV	2331413	NA	P	Chromosomal deletion spanning introns and exons is undetectable by ES
44	Female	Deafness–infertility syndrome	*STRC; CATSPER2*	Hom	NC_000015.9:g.43873201_43940259del	CNV	20301780	NA	P	Interfered by the pseudogene, analyzing this region via ES data is challenging

Abbreviations: ACMG/AMP, American College of Medical Genetics and Genomics/Association for Molecular Pathology; Comp het, compound heterozygous; DFNB1A, deafness autosomal recessive 1A; Het, heterozygous; HGVS, human genome variation society; Hom, homozygous; LP, likely pathogenic; NA, not applicable; P, pathogenic.

## Data Availability

All data supporting the findings of this study are available either within the article, supplementary material, or from the corresponding authors upon reasonable request. The data are not publicly available due to privacy or ethical restrictions.

## Supporting Information

Supporting Information Additional supporting information can be found online in the Supporting Information section. Supporting Information Table S1. Demographic characteristics of 46 probands of Cohort 1. Table S2. Reinterpretation of two *LARS2* variants. Figure S1. A homozygous and heterozygous deletion of *ADGRV1* in Family 28. Figure S2. A de novo 3.7 Mb deletion in Family 42. Table S3. Genotype and phenotype of seven patients harboring a heterozygous DFNB1 deletion. Table S4. Haplotype analysis of the novel del(125 kb). Table S5. Summary of DFNB1 deletions reported in other studies.
