# Supplementary material for: Genome Sequencing Unveils the Role of Copy Number Variants in Hearing Loss and Identifies Novel Deletions With Founder Effect in the DFNB1 Locus
Source: Hum Mutat. 2024 Aug 6;2024:9517114. doi: 10.1155/2024/9517114 (PMC11918852; doi:10.1155/2024/9517114)
Supplement: Supporting Information — Additional supporting information can be found online in the Supporting Information section. Supporting Information Table S1. Demographic characteristics of 46 probands of Cohort 1. Table S2. Reinterpretation of two LARS2 variants. Figure S1. A homozygous and heterozygous deletion of ADGRV1 in Family 28. Figure S2. A de novo 3.7 Mb deletion in Family 42. Table S3. Genotype and phenotype of seven patients harboring a heterozygous DFNB1 deletion. Table S4. Haplotype analysis of the novel del(125 kb). Table S5. Summary of DFNB1 deletions reported in other studies. [file 9517114.f1.docx]

**Table S1** Demographic characteristics of 46 probands of cohort 1

| **Characteristic** | **No.** | **Percentage (%)** |
| --- | --- | --- |
| **All** | 46 | 100 |
| **Sex** |  |  |
| Male | 24 | 52 |
| Female | 22 | 48 |
| **Family history** |  |  |
| Yes | 12 | 26 |
| No | 34 | 74 |
| **Newborn hearing screening** |  |  |
| Pass | 10 | 22 |
| referral | 28 | 61 |
| Not tested | 8 | 17 |
| **Onset** |  |  |
| Prelingual | 43 | 93 |
| Post-lingual | 3 | 7 |
| **Severity** |  |  |
| Mild | 4 | 9 |
| Moderate | 11 | 24 |
| Severe | 5 | 11 |
| Profound | 26 | 57 |
| **Rehabitation** |  |  |
| Hearing aids | 12 | 26 |
| cochlear implants | 14 | 30 |
| Both | 2 | 4 |
| Not applied | 18 | 39 |

**Table S2** Reinterpretation of two *LARS2* variants

| Gene | HGVS variant | Initial ACMG/AMP criteria | Initial classification | Reasons for reclassification | New ACMG/AMP  criteria | New classification |
| --- | --- | --- | --- | --- | --- | --- |
| *LARS2* | NC_000003.11:g.45557610C>T  NM_015340.4:c.1886C>T | PM2, PM3, PP4 | VUS | New literature (PMID:34997062) | PM2, PM3, PP4, PP1 | LP |
| *LARS2* | NC_000003.11:g.45541972T>C  NM_015340.4:c.1661T>C | PM2 | VUS | Two probands detected in this study | PM2, PM3_Strong | LP |

Abbreviation: VUS, variant of uncertain significance.

**Figure S1** A homozygous and heterozygous deletion of *ADGRV1* in family 28. **A** Pedigree of family 28. The proband and his sister carried a homozygous 6.8 kb deletion of *ADGRV1*, presenting with hearing loss. Their parents had a heterozygous deletion of *ADGRV1*, and exhibited normal hearing. **B** Schematic illustration of the deletion in the *ADGRV1* gene. **C** CNV analysis of *ADGRV1* using ES data. The arrow indicates the copy ratio of exon 87 of *ADGRV1*. The homozygous deletion of exon 87 of *ADGRV1* was successfully called using ES data (left arrow), while the heterozygous deletion failed to be detected using ES data (right arrow). **D** CNV analysis of *ADGRV1* using GS data. Both the homozygous and heterozygous deletion of *ADGRV1* were successfully identified using GS data.

**Figure S2** A *de novo* 3.7 Mb deletion in family 42. **A** Pedigree of family 42. The proband carried a *de novo* 3.7 Mb deletion in chromosome 17p11.2 and was diagnosed with Smith-Magenis Syndrome. In addition to hearing loss, this proband exhibited developmental delay. **B** CNV analysis of the 3.7 Mb deletion in 17p11.2 using GS data. **C** Schematic illustration of the 3.7 Mb deletion.

**Table S3** Genotype and phenotype of seven patients harboring a heterozygous DFNB1 deletion

| Patient ID | Gender | Age | Gene | Zygosity | HGVS variant | Newborn hearing screening | Onset | Severity |
| --- | --- | --- | --- | --- | --- | --- | --- | --- |
| P10 | Male | 4 | *GJB2* | Het | NM_004004.6:c.109G>A | Referral | Prelingual | Moderate |
|  |  |  | - | Het | NC_000013.10:g.20972509_21097962del |  |  |  |
| P34 | Male | 2 | *GJB2* | Het | NM_004004.6:c.109G>A | Referral | Prelingual | Mild |
|  |  |  | - | Het | NC_000013.10:g.20972509_21097962del |  |  |  |
| P43 | Female | 11 | *GJB2* | Het | NM_004004.6:c.299_300del | Referral | Prelingual | Profound |
|  |  |  | - | Het | NC_000013.10:g.20802727_21034768del |  |  |  |
| P66 | Female | 7 | *GJB2* | Het | NM_004004.6:c.109G>A | Referral | Prelingual | Mild |
|  |  |  | - | Het | NC_000013.10:g.20972509_21097962del |  |  |  |
| P71 | Female | 2 | *GJB2* | Het | NM_004004.6:c.109G>A | Referral | Prelingual | Moderate |
|  |  |  | - | Het | NC_000013.10:g.20972509_21097962del |  |  |  |
| P76 | Female | 5 | *GJB2* | Het | NM_004004.6:c.109G>A | Referral | Prelingual | Moderate |
|  |  |  | - | Het | NC_000013.10:g.20972509_21097962del |  |  |  |
| P82 | Male | 7 | *GJB2* | Het | NM_004004.6:c.235del | Referral | Prelingual | Profound |
|  |  |  | - | Het | NC_000013.10:g.20972509_21097962del |  |  |  |

**Table S4** Haplotype analysis of the novel del(125 kb)

| Marker | Position (hg19) | Distance from del(125 kb) | Allele frequency  (ChinaMap) | | Allele frequency  (this study) | | *p* value |
| --- | --- | --- | --- | --- | --- | --- | --- |
| rs967521 | chr13:20508699 | 463809 | A:12279 | G:8897 | A:4 | G:4 | *p* = 0.728 |
| [rs9552058](https://www.ncbi.nlm.nih.gov/projects/SNP/snp_ref.cgi?searchType=adhoc_search&type=rs&rs=rs9552058) | chr13:20569878 | 402630 | A:6438 | T:14738 | A:3 | T:5 | *p* = 0.706 |
| rs7998272 | chr13:20627310 | 345198 | A:6357 | G:14819 | A:3 | G:5 | *p* = 0.704 |
| rs4769938 | chr13:20646473 | 326035 | C:7161 | T:14015 | C:3 | T:5 | *p* > 0.999 |
| rs9579781 | chr13:20674373 | 298135 | T:4344 | C:16832 | T:3 | C:5 | *p* = 0.215 |
| rs57902484 | chr13:20711744 | 260764 | T:11219 | C:9957 | T:5 | C:3 | *p* = 0.730 |
| rs3751385 | chr13:20762956 | 209552 | A:9685 | G:11491 | A:4 | G:4 | *p* > 0.999 |
| rs7339123 | chr13:20810986 | 161522 | T:7688 | C:13488 | T:2 | C:6 | *p* = 0.719 |
| rs9550626 | chr13:20861153 | 111355 | C:13609 | G:7567 | C:7 | G:1 | *p* = 0.273 |
| rs60084509 | chr13:20910450 | 62058 | C:11546 | A:9630 | C:2 | A:6 | *p* = 0.153 |
| [rs4238153](https://www.ncbi.nlm.nih.gov/projects/SNP/snp_ref.cgi?searchType=adhoc_search&type=rs&rs=rs4238153) | chr13:20971841 | 667 | C:5447 | T:15729 | C:8 | T:0 | *p* <0.01 |
| del(125 kb) | chr13:20972508-21097961 | 0 | del | del | del | del | / |
| rs61955540 | chr13:21099598 | 1637 | C:13540 | T:7636 | C:8 | T:0 | *p* = 0.057 |
| [rs2313975](https://www.ncbi.nlm.nih.gov/projects/SNP/snp_ref.cgi?searchType=adhoc_search&type=rs&rs=rs2313975) | chr13:21121698 | 23737 | A:7960 | G:13216 | A:0 | G:8 | *p* = 0.029 |
| [rs9509284](https://www.ncbi.nlm.nih.gov/projects/SNP/snp_ref.cgi?searchType=adhoc_search&type=rs&rs=rs9509284) | chr13:21146232 | 48271 | A:6764 | G:14412 | A:7 | G:1 | *p* < 0.01 |
| [rs76606061](https://www.ncbi.nlm.nih.gov/projects/SNP/snp_ref.cgi?searchType=adhoc_search&type=rs&rs=rs76606061) | chr13:21189487 | 91526 | T:14074 | C:7102 | T:1 | C:7 | *p* < 0.01 |
| [rs9552255](https://www.ncbi.nlm.nih.gov/projects/SNP/snp_ref.cgi?searchType=adhoc_search&type=rs&rs=rs9552255) | chr13:21241823 | 143862 | C:13937 | T:7239 | C:8 | T:0 | *p* = 0.058 |
| [rs2772175](https://www.ncbi.nlm.nih.gov/projects/SNP/snp_ref.cgi?searchType=adhoc_search&type=rs&rs=rs2772175) | chr13:21300712 | 202751 | T:5966 | A:15210 | T:7 | A:1 | *p* < 0.01 |
| [rs7995798](https://www.ncbi.nlm.nih.gov/projects/SNP/snp_ref.cgi?searchType=adhoc_search&type=rs&rs=rs7995798) | chr13:21338432 | 240471 | C:6188 | T:14988 | C:7 | T:1 | *p* < 0.01 |
| [rs77515877](https://www.ncbi.nlm.nih.gov/projects/SNP/snp_ref.cgi?searchType=adhoc_search&type=rs&rs=rs77515877) | chr13:21484852 | 386891 | G:11534 | A:9642 | G:6 | A:2 | *p* = 0.305 |
| rs76206105 | chr13:21542182 | 444221 | C:10229 | G:10947 | C:4 | G:4 | *p* > 0.999 |
| rs11840914 | chr13:21590089 | 492128 | C:10237 | T:10939 | C:4 | T:4 | *p* > 0.999 |

**Table S5** Summary of DFNB1 deletions reported in other studies

| DFNB1 deletion | Genetic ancestry group | Reference  (PMID) | Case number of patients  with hearing loss |
| --- | --- | --- | --- |
| del(*GJB6*-D13S1830) | European | 14571368 | 102 |
| del(*GJB6*-D13S1854) | European | 15994881 | 18 |
| del(101 kb) | European | 28405014 | 11 |
| del(131 kb) | European | 20236118 | 4 |
| del(>920 kb) | European | 19101659 | 1 |
| del(179 kb) | European | 26444186 | 1 |
